# Report on Dental Physiology

**Published:** 1876-05

**Authors:** D. C. Hauxhurst


### REPORT ON DENTAL PHYSIOLOGY.

BY D. C. HAUXHURST.

*Read Before the Michigan State Dental Society, March 29, 1876.*

The earlier stages of dental development constitute a most difficult and intricate subject of investigation. I believe it is far easier to get a fair knowledge of dentine, enamel, cementum and tooth pulp, as found in the mature tooth, than to get anything like an adequate knowledge of the progressive changes through which the tooth germ must pass during gestation, and afterwards, in order to become the perfect tooth. The building of a tooth in all its perfection, involves the laying down of dense tissues from fluid or semi fluid pabulum, by complex physiological processes, which not only affects the jaws and the fifth nerve seriously, but capable of profoundly modifying vital action in every part of the organism, while they are in progress. Hence, it becomes us as professional men, to put our students through a thorough course of dental embryology.

It is certainly important to the student to have accurate ideas of the nature, origin and progressive development of the germs of teeth. And nothing will so much facilitate this as the actual dissection and study of these germs, from the time when they are first recognizable to the end of gestation, and indeed to the time of their eruption. And this is not so formidable an undertaking as might be inferred; I believe it to be quite in the power of every one.

To this end it is necessary to obtain a considerable number of fœtal jaws in all stages of development.

To study the developing teeth from books and from books alone, is better than not to study at all. But in this way, only a delusive and uncertain knowledge of the subject can be obtained. A real acquaintance with those formative stages through which the teeth must pass before they become the completed organs that we operate upon, can be best obtained by an examination of young jaws.

The real tissues must meet the eye; the fingers must handle and pull them to pieces; the delicate saculi must be turned out of their osseous beds; the young pulps must be hardened by re-agents and sliced for investigation, or mashed between the fingers by way of learning their consistency. If one of them happens to burst and discharge its contents into the face it is all the better; the accident will impress the fact that teeth in their earlier stages are more like soft cysts of organic matter than like teeth. At any rate the real tissues must be procured and examined by every method that can help to reveal their characteristics.

I know of no better way to procure a supply of fœtal jaws than to engage some honest butcher to save all the embryos that he meets with for say three months. This he will willingly do for a small sum, since they are a kind of stock that is not usually regarded as valuable. He will send them up undisturbed and completely protected by their natural membranes and the uterus. If you desire to inject the vessels preparatory to a study of the distribution of capilaries in the young pulps you will pay him a small sum extra for bringing the specimen warm, as the injecting fluid is best thrown in before the rigidity of death has come on.

A few weeks or months will furnish an abundance of material. The young jaws will be cut out and either examined immediately or put into bottles or jars and set away for future examination. In the latter case they must be carefully labeled with the name of the animal from which taken, the proximate age and the preserving fluid used. It is best to have good large labels or tickets, so as to give room for the successive dates at which the specimens are removed from one re-agent and subjected to that of another.

In general it will be better for the student to dissect several—a good many—of these embryonic jaws, noting all the physical characteristics before passing to the minute anatomy of the young pulp with the microscope. Curved needles may be set in the stocks of old excavators to facilitate the examination.

If those specimens which are designed, for a first rough dissection, be subjected to the action of alcohol for a week or two, the pulpy mass which occupies the seat of the future tooth, will be measureably hardened, and may be more easily taken from its osseous, or cartilaginous cavity. If a crystal of hematoxylon be thrown into the alchohol used in hardening, the more recently formed tissues will be stained most deeply, and anatomical peculiarities will be brought out, that would escape notice by the ordinary method.

If the osseous walls that enclose the dental saculi are hard enough to interpose an obstacle to easy examination, the jaws may be thrown into a five per cent solution of hydrochloric acid water for a time, and then subjected to the influence of alcohol as above, thus the bony tissue will be rendered soft and manageable.

I have found this preliminary decalcification particularly desirable to facilitate the study of the dental germs in the jaws of calves, directly after birth and from that time onward. The alveolar walls may be sufficiently softened, so that they are easily sliced up, and the young germs exposed, or turned out from their bed, under the milk set. For these specimens, however, I prefer a little additional treatment.

After softening with acid, they should be subjected to a 1 to 3 per cent solution of chromic acid in water, and then transferred to alcohol. By this means a very manageable specimen will be produced, and one which by the aid of a pocket lens, a pair of needles set in handles, a jack knife, a razor, a copy of Gray's Anatomy, a pair of good eyes, and a good head behind them, will be capable of yielding the student a kind of practical acquaintance with the process of tooth formations, far superior to the vague and dim conceptions derived from merely reading about these things in a book.

If dentists studied more carefully these processes they would better understand how to manage cases of teething. They would manipulate with more care not to disturb the germs under the milk teeth. They would understand better how to regulate unevenly developed teeth. They would acquire sharper conceptions of how the mineral constituents of the teeth are carried to their destination by the blood, and deposited in the cell-matrix into dentine by the odontoblasts on into the enamel by the enamel membrane. They would the better appreciate the importance of preserving the highest degree of health of the mother during gestation, and of both mother and child during lactation, and of the child in particular during the earlier years while the teeth are in their formative stages.

